# Changes in Cancer Screening in the US During the COVID-19 Pandemic

**DOI:** 10.1001/jamanetworkopen.2022.15490

**Published:** 2022-06-03

**Authors:** Stacey A. Fedewa, Jessica Star, Priti Bandi, Adair Minihan, Xuesong Han, K. Robin Yabroff, Ahmedin Jemal

**Affiliations:** 1 Surveillance and Health Equity Sciences, American Cancer Society, Atlanta, Georgia; 2 Now with Department of Hematology and Oncology, Emory University, Atlanta, Georgia

## Abstract

**Importance:**

Health care was disrupted in the US during the first quarter of 2020 with the emergence of the COVID-19 pandemic. Early reports in selected samples suggested that cancer screening services decreased greatly, but population-based estimates of cancer screening prevalence during 2020 have not yet been reported.

**Objective:**

To examine changes in breast cancer (BC), cervical cancer (CC), and colorectal cancer (CRC) screening prevalence with contemporary national, population-based Behavioral Risk Factor Surveillance System (BRFSS) data.

**Design, Setting, and Participants:**

This survey study included respondents from the 2014, 2016, 2018, and 2020 BRFSS surveys who were eligible for BC (women aged 50-74 years), CC (women aged 25-64 years), and CRC (women and men aged 50-75 years) screening. Data analysis was performed from September 2021 to February 2022.

**Exposures:**

Calendar year.

**Main Outcomes and Measures:**

Self-reported receipt of a recent (defined as in the past year) BC, CC, and CRC screening test. Adjusted prevalence ratios (aPRs) comparing 2020 vs 2018 prevalence and 95% CIs were computed.

**Results:**

In total, 479 248 individuals were included in the analyses of BC screening, 301 453 individuals were included in CC screening, and 854 210 individuals were included in CRC screening, In 2020, among respondents aged 50 to 75 years, 14 815 (11.4%) were Black, 12 081 (12.6%) were Hispanic, 156 198 (67.3%) were White, and 79 234 (29.9%) graduated from college (all percentages are weighted). After 4 years (2014-2018) of nearly steady prevalence, past-year BC screening decreased by 6% between 2018 and 2020 (from 61.6% in 2018 to 57.8% in 2020; aPR, 0.94; 95% CI, 0.92-0.96), and CC screening decreased by 11% (from 58.3% in 2018 to 51.9% in 2020; aPR, 0.89; 95% CI, 0.87-0.91). The magnitude of these decreases was greater in people with lower educational attainment and Hispanic persons. CRC screening prevalence remained steady; past-year stool testing increased by 7% (aPR, 1.07; 95% CI, 1.02-1.12), offsetting a 16% decrease in colonoscopy (aPR, 0.84; 95% CI, 0.82-0.88) between 2018 and 2020.

**Conclusions and Relevance:**

In this survey study, stool testing increased and counterbalanced a decrease in colonoscopy during 2020, and BC and CC screening modestly decreased. How these findings might be associated with outcomes is not yet known, but they will be important to monitor, especially in populations with lower socioeconomic status, who experienced greater screening decreases during the COVID-19 pandemic.

## Key Points

**Question:**

Did the national prevalence of breast, cervical, and colorectal cancer screening change during the COVID-19 pandemic?

**Findings:**

In this national survey study, between 2018 and 2020, past-year breast and cervical cancer screening prevalence declined by 6% and 11%, respectively. There was no change in past-year colorectal cancer screening prevalence, with a 7% increase in stool testing and a 16% decrease in colonoscopy.

**Meaning:**

These findings suggest that stool testing counterbalanced decreases in colonoscopy during 2020, whereas breast and cervical cancer screening decreased modestly.

## Teaser

This survey study examines changes in breast, cervical, and colorectal cancer screening prevalence during the COVID-19 pandemic in the US.

## Introduction

The emergence of the COVID-19 pandemic in the first quarter of 2020 caused disruptions in health care utilization in the US.^1,2^ Stay-at-home orders were issued, and professional societies recommended pausing routine cancer screening tests. Several reports based on commercially or Medicare insured adults note sharp decreases (80%-90%) in breast cancer (BC), cervical cancer (CC), and colorectal cancer (CRC) screening volumes in March and April of 2020.^2,3,4,5^ After the implementation of safety protocols and reopening of cancer screening services, screening volumes improved to prepandemic levels during the summer of 2020, but perhaps not enough to overcome previous decreases.^3^

The magnitude of potential deficits and population-based estimates of cancer screening during 2020 are not yet known, because previous studies^3,4,5,6,7,8,9^ using medical claims and records, from which pandemic-related screening disruptions have been measured, were based on restricted geographical regions or were conducted among people who maintained the same health insurance coverage throughout 2020. Furthermore, those previous studies^3,4,5,6,7,8,9^ were not able to examine both recent and guideline-concordant screening because medical claims often do not contain a person’s longer-term screening history. Both are important to measure because short-term screening practices may be more closely linked to health care disruptions, whereas adherence to longer screening intervals may be more closely associated with cancer outcomes. It is also unknown whether any changes in population-wide screening were worse among populations who have historically faced barriers to accessing health care and may be especially vulnerable to health care disruptions.^10^

In this survey study, we examined recent and guideline-recommended BC, CC, and CRC screening prevalence, with population-based 2020 Behavioral Risk Factor Surveillance System (BRFSS) data and compared it with prior years. We also examined whether potential changes in cancer screening prevalence varied according to race, ethnicity, and socioeconomic status (SES) to assess whether inequities in screening worsened.

## Methods

This was a survey study based on 2014, 2016, 2018, and 2020 BRFSS data, an annual state-based telephone survey overseen by the Centers for Disease Control and Prevention, with respective response rates of 47.0%, 47.0%, 49.9%, and 47.9%.^11^ The study was based on deidentified publicly available data, which the US Department of Health and Human Services considers as nonhuman research and does not require institutional review board review or informed consent.^12^ The National Health Center for Statistics data suppression guidance was followed.^13^

In even-numbered survey years, respondents were asked whether they have ever had a specific screening test and when their most recent test occurred (eTable 1 in the Supplement). BRFSS conducts interviews throughout the year and, in 2020, the national proportions of interviews conducted per month were comparable to those in previous years. However, several states halted interviews beginning in March 2020 and, as such, we focused on national estimates (eTable 2 in the Supplement).

The primary outcomes were self-reported receipt of BC screening (mammography), CC screening (Papanicolaou and human papillomavirus testing [HPV]), and CRC screening (colonoscopy, stool testing, stool DNA, computed tomography colonography, or sigmoidoscopy) in the past year. We focused on past-year screening because health care disruptions may be more closely associated with recent screening testing. We also considered whether participants were up to date (UTD) with US Preventive Services Task Force (USPSTF) recommended cancer screening, which have longer intervals (eg, colonoscopy every 10 years for CRC screening; see eTable 1 in the Supplement), because being UTD is more closely associated with cancer outcomes. Prostate-specific antigen testing for prostate cancer screening was not considered because it is recommended only with shared decision-making.^14^

Primary analyses were restricted to the following age ranges to align with USPSTF recommendations: 50 to 74 years for BC, 25 to 64 years for CC, and 50 to 75 years for CRC screening. Respondents with cancer or who were missing cancer screening data (BC, 32 219 individuals [6.3%]; CC, 47 013 individuals [8.1%]; and CRC, 77 832 individuals [8.4%]) were excluded from the analyses (eTable 3 in the Supplement).

Covariates were selected on the basis of previously reported associations with cancer screening and included self-reported race and ethnicity (ie, American Indian or Alaska Native, Asian or Pacific Islander, Hispanic, non-Hispanic Black, non-Hispanic White, and other [ie, not otherwise specified]), age, sex (for CRC only), annual household income, educational attainment, having a usual source of care, and a primary care visit in the past 12 months.^15,16,17^ Employment status and insurance coverage (yes vs no) were considered among people younger than 65 years.

### Statistical Analysis

 Data analysis was performed from September 2021 to February 2022. Past year and UTD screening prevalence were examined by year. Probabilities calculated from logistic regression models were used to estimate unadjusted and adjusted prevalence ratios (aPRs) and 95% CIs.^18^ To determine whether inequities in screening worsened, changes in screening prevalence according to sociodemographic and socioeconomic factors were assessed. In sensitivity analyses, aPRs comparing screening in April to December of 2020, after the onset of the pandemic, vs 2018 were computed. To determine whether changes during 2020 and specifically in April to December were part of ongoing seasonal trends, prevalence according to interview quarters as computed. All estimates used Centers for Disease Control and Prevention–recommended weights to be nationally representative and account for nonresponse. SAS statistical software version 9.4 (SAS Institute) was used for analyses. Significance (*P* < .05) was determined with 2-sided χ^2^ tests.

## Results

### Respondent Characteristics

In total, 479 248 individuals were included in the analyses of BC, 301 453 individuals were included in the analyses for CC, and 854 210 individuals were included in the analyses for CRC screening. In 2020, among respondents aged 50 to 75 years, 14 815 (11.4%) were Black, 12 081 (12.6%) were Hispanic, 156 198 (67.3%) were White, and 79 234 (29.9%) graduated from college (all percentages are weighted). In terms of health care access and utilization, 10.5% of nonelderly respondents (9890 individuals) were uninsured, and most respondents reported a primary care practitioner visit and usual source of care, similar to 2018 proportions (Table 1). Analogous patterns were observed for persons eligible for BC and CC screening.

**Table 1.** Characteristics of Screening Eligible Adults According to Survey Year

Characteristic	Respondents, No. (weighted %)								
	Adults eligible for CRC screening (aged 50-75 y)			Women eligible for breast cancer screening (aged 50-74 y)			Women eligible for cervical cancer screening (aged 25-64 y)		
	2018 (n = 226 106)	2020 (n = 200 075)	*P* value^a^	2018 (n = 120 674)	2020 (n = 106 389)	*P* value^a^	2018 (n = 100 828)	2020 (n = 91 491)	*P* value^a^
Sex									
Female	125 100 (52.2)	109 934 (52.0)	.63	NA	NA	NA	NA	NA	NA
Male	100 423 (47.8)	90 141 (48.0)		NA	NA		NA	NA	
Age, y									
25-39	NA	NA	.003	NA	NA	.01	31 588 (41.3)	29 731 (42.0)	.27
40-49	NA	NA		NA	NA		23 098 (24.1)	22 034 (24.1)	
50-54	37 505 (23.0)	34 035 (22.3)		20 302 (23.1)	18 405 (22.1)		14 068 (12.5)	12 436 (11.9)	
55-59	42 613 (20.6)	36 505 (20.1)		23 203 (20.8)	19 649 (20.7)		15 363 (10.8)	12 981 (10.9)	
60-64	47 982 (22.0)	41 584 (22.0)		26 219 (22.6)	22 462 (22.2)		16 711 (11.3)	14 309 (11.1)	
65-69	49 319 (17.4)	43 362 (17.7)		27 464 (17.9)	23 960 (18.3)		NA	NA	
70-75	48 687 (17.1)	44 589 (18.0)		23 486 (15.6)	21 913 (16.7)		NA	NA	
Race and ethnicity									
American Indian or Alaska Native	3802 (0.9)	3316 (1.0)	<.001	2059 (0.9)	1818 (0.9)	.009	2046 (0.9)	1822 (1.0)	.009
Asian or Pacific Islander^b^	4037 (3.8)	4034 (3.8)		1985 (3.6)	2062 (3.4)		3146 (6.0)	3360 (6.4)	
Hispanic	12 558 (11.7)	12 081 (12.6)		6701(11.4)	6501 (12.5)		11061 (18.6)	10 899 (19.8)	
Non-Hispanic Black	18 121 (10.6)	14 815 (11.4)		10 757 (11.2)	8624 (11.6)		9427 (12.4)	7809 (12.3)	
Non-Hispanic White	177 663 (69.4)	156 198 (67.3)		94 493 (69.6)	82 674 (67.8)		70 953 (58.7)	63 124 (56.9)	
Other^c^	5251 (1.4)	4856 (1.6)		2544 (1.3)	2432 (1.6)		2692 (1.7)	2877 (2.0)	
Household income, $									
<25 000	46 721 (25.5)	36 704 (23.8)	<.001	26 424 (27.7)	20 574 (25.2)	<.001	21 174 (25.8)	17 260 (23.9)	.001
25 to <75 000	75 539 (37.5)	63 502 (36.8)		40 172 (37.9)	34 219 (38.8)		32 260 (35.1)	28 593 (35.4)	
≥75 000	65 683 (37.0)	60 644 (39.3)		31 075 (34.4)	28 662 (35.9)		34 462 (39.1)	32 665 (40.7)	
Education									
Less than high school	15 434 (13.3)	12 069 (12.5)	<.001	7639 (12.5)	5897 (11.7)	.002	6299 (11.3)	5108 (11.1)	.06
High school	60 486 (26.9)	51 701 (26.3)		31 616 (26.3)	26 419 (25.8)		21 425 (21.7)	18 857 (21.3)	
Some college	62 112 (30.7)	56 062 (30.7)		34 980 (32.5)	31 325 (32.1)		27 338 (30.6)	24 949 (30.1)	
College	87 185 (28.7)	79 234 (29.9)		46 024 (28.4)	42 230 (29.8)		45 546 (36.3)	42 298 (37.1)	
Recent primary care practitioner visit	191 039 (84.1)	165 608 (81.9)	<.001	103 579 (85.6)	89 709 (83.3)	<.001	77 539 (76.1)	68 561 (73.1)	<.001
Usual source of care	202 007 (88.6)	178 309 (88.3)	.37	110 466 (91.1)	97 322 (90.7)	.28	83 464 (79.9)	75 304 (78.6)	.005
Uninsured (age <65 y)	10 666 (10.1)	9890 (10.5)	.23	5090 (9.0)	4729 (9.1)	.85	10 159 (12.9)	9561 (13.6)	.06
Employment status (age <65 y)									
Employed	79 353 (62.6)	69 866 (62.5)	<.001	40 169 (56.9)	35 116 (56.9)	<.001	67 699 (66.5)	61 300 (64.7)	<.001
Unemployed >1 y	3442 (3.2)	2772 (2.8)		1792 (3.3)	1447 (2.7)		2582 (3.0)	2274 (3.0)	
Unemployed <1 y	25 63 (2.2)	4911 (4.8)		1383 (2.1)	2664 (5.0)		2587 (2.7)	5236 (6.7)	
Not in labor force	23 332 (18.0)	19 023 (17.8)		15 178 (22.5)	12 311 (22.2)		18 660 (20.2)	15 134 (18.5)	
Unable to work	17 926 (14.1)	13 408 (12.1)		10 426 (15.3)	7844 (13.2)		8530 (7.6)	6732 (7.0)	

Abbreviation: NA, not applicable.

^a^ *P* values comparing 2018 and 2020 data were calculated with χ^2^ statistics.

^b^ Includes both Hispanic and non-Hispanic individuals.

^c^ Other refers to race only, non-Hispanic, not otherwise specified.

### Past-Year Screening Trends

Between 2018 and 2020, past-year BC screening decreased by 6% between 2018 and 2020 (from 61.6% in 2018 to 57.8% in 2020; aPR, 0.94; 95% CI, 0.92-0.95), and CC screening decreased by 11% (from 58.3% in 2018 to 51.9% in 2020; aPR, 0.89; 95% CI, 0.87-0.91) after 4 previous years (2014-2018) of mostly stable BC and CC screening prevalence (Figure and Table 2). In 2020, an estimated 2.13 and 4.47 million fewer women reported receiving BC and CC screening, respectively, in the past year than in 2018 (eFigure 1 in the Supplement). Decreases in 2020 past-year BC and CC screening prevalence were largely associated with decreases beginning in the third quarter of 2020, a seasonal pattern that was not observed in previous years (Figure). Past-year screening prevalence during April through December 2020 was 8% (aPR, 0.92; 95% CI, 0.90-0.94) lower for BC and 12% (aPR, 0.88; 95% CI, 0.86-0.90) lower for CC than 2018 estimates (eTable 4 in the Supplement).

**Figure.**
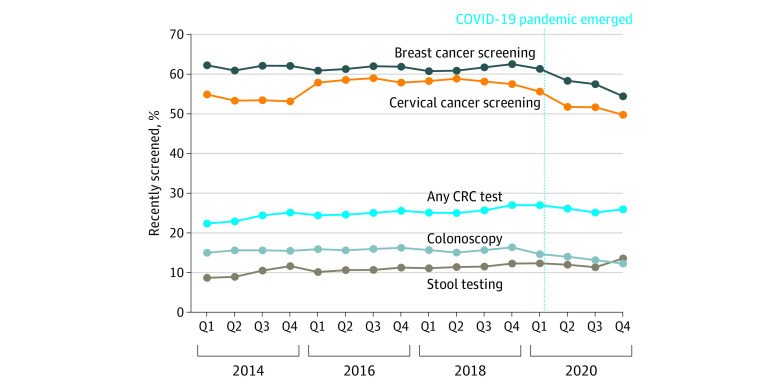
Past-Year Breast Cancer, Cervical Cancer, and Colorectal Cancer (CRC) Screening According to Interview Quarter (Q) and Year, Behavioral Risk Factor Surveillance System, 2014-2020

**Table 2.** Unadjusted and Adjusted Prevalence and PRs of Recent Cancer Screening According to Year, Behavioral Risk Factor Surveillance System 2014, 2016, 2018, and 2020

Prevalence type	Prevalence, %				PR (95% CI)		
	2014	2016	2018	2020	2016 vs 2014	2018 vs 2016	2020 vs 2018
Unadjusted							
Breast	62.0	61.7	61.6	57.8	1.00 (0.98-1.01)	1.00 (0.98-1.01)	0.94 (0.92-0.96)
Cervical	53.8	58.5	58.3	51.9	1.09 (1.07-1.10)	1.00 (0.98-1.01)	0.89 (0.87-0.91)
Any CRC testing	23.7	24.9	25.7	25.9	1.05 (1.03-1.07)	1.03 (1.01-1.05)	1.01 (0.98-1.04)
Colonoscopy	15.4	15.8	15.6	13.2	1.03 (1.00-1.06)	0.99 (0.96-1.01)	0.85 (0.82-0.88)
Stool testing	9.9	10.6	11.5	12.3	1.07 (1.03-1.11)	1.08 (1.04-1.13)	1.07 (1.03-1.12)
Adjusted^a^							
Breast	62.3	61.8	61.5	57.5	0.99 (0.98-1.00)	0.99 (0.98-1.01)	0.94 (0.92-0.95)
Cervical	53.9	58.4	58.3	51.9	1.08 (1.07-1.10)	1.00 (0.98-1.02)	0.89 (0.87-0.91)
Any CRC testing	24.1	25.0	25.5	25.6	1.03 (1.01-1.06)	1.02 (1.00-1.05)	1.00 (0.98-1.03)
Colonoscopy	15.4	15.9	15.6	13.2	1.03 (1.00-1.06)	0.98 (0.95-1.01)	0.84 (0.82-0.87)
Stool testing	10.2	10.6	11.4	12.1	1.04 (1.00-1.08)	1.07 (1.03-1.11)	1.07 (1.02-1.12)

Abbreviations: CRC, colorectal cancer; PR, prevalence ratio.

^a^ Models were adjusted for age, sex (CRC screening), state, and education.

For CRC screening, past-year colonoscopy prevalence decreased by 16% (from 15.6% to 13.2%; aPR, 0.84; 95% CI, 0.82-0.87), whereas the prevalence of stool testing increased by 7% (from 11.4% to 12.1%; aPR, 1.07; 95% CI, 1.02-1.12) between 2018 and 2020 (Table 2). Sigmoidoscopy was rarely used (2018 prevalence, 0.6%; 2020 prevalence, 1.1%). In 2020, 2.9% of respondents reported a recent sDNA test, and 1.1% reported a recent computed tomography colonography. There was no change in past-year CRC screening between 2018 and 2020 when sDNA and computed tomography colonography were considered (aPR, 1.02; 95% CI, 0.99-1.04) or when these 2 tests were excluded from 2020 estimates (aPR, 0.90; 95% CI, 0.95-1.00).

### Changes in Past-Year Screening According to Sociodemographic and Socioeconomic Groups

In 2020, past-year BC and CC screening prevalence rates were lower in people with less than a high school diploma compared with those with college degrees (BC, 50.3% vs 62.2%; CC, 43.6% vs 55.7%) (Table 3). In addition, between 2018 and 2020, past-year BC screening prevalence decreased by 10% (aPR, 0.90; 95% CI, 0.83-0.97) and CC screening prevalence decreased by 17% (aPR, 0.83; 95% CI, 0.75-0.91) among people with less than a high school diploma compared with a 6% decrease for BC (aPR, 0.94; 95% CI, 0.91-0.96) and a 9% decrease for CC (aPR, 0.91; 95% CI, 0.88-0.93) among college-educated respondents (Table 4). Past-year stool testing was an exception to this pattern, where there was no change for college graduates, and people with less than a high school diploma reported a striking 39% increase between 2018 and 2020 (aPR, 1.39; 95% CI, 1.20-1.62) (Table 4). However, overall CRC screening utilization remained lower in people with lower educational attainment (Table 3).

**Table 3.** Unadjusted Prevalence of Recent (Past-Year) Cancer Screening According to Sociodemographic, Socioeconomic, and Access to Care Variables, Behavioral Risk Factor Surveillance System, 2018 and 2020

Variable	Breast (women aged 50-74 y)			Cervical (aged women 25-64 y)			Any CRC test (adults aged 50-75 y)			Colonoscopy (adults aged 50-75 y)			Stool testing (adults aged 50-75 y)		
	Prevalence, %		*P* value	Prevalence, %		*P* value	Prevalence, %		*P* value	Prevalence, %		*P* value	Prevalence, %		*P* value
	2018	2020		2018	2020		2018	2020		2018	2020		2018	2020	
Total	61.6	57.8	<.001	58.3	52.1	<.001	25.7	26	.42	15.6	13.4	<.001	11.5	12.3	.003
Race and ethnicity															
American Indian or Alaska Native	55.4	48.4	.12	53.6	52.1	.76	25.3	24.3	.71	15.2	11.8	.09	12	12.3	.87
Asian or Pacific Islander	63.9	46.6	.001	48.8	44.8	.21	29.6	24.6	.12	12.6	8.4	.01	18.6	15.1	.25
Hispanic	59.5	53.2	.006	57.1	47.4	<.001	24	29.6	<.001	12.8	11.2	.10	12.3	19	<.001
Non-Hispanic Black	67.6	65.5	.19	67.2	62.4	.001	29.4	29.9	.67	18.5	16.5	.03	13.8	14	.81
Non-Hispanic White	61.1	58.4	<.001	58	51.9	<.001	25.2	24.8	.32	15.9	13.5	<.001	10.6	10.7	.54
Other^a^	56.6	47.4	.01	52.6	48.6	.18	28.2	23.5	.03	15	10.1	<.001	14.5	12.8	.39
Education															
Less than high school	56.5	50.3	.005	53	43.6	<.001	24.8	28.8	.003	13.7	11.3	.009	12.1	16.9	<.001
High school	59.8	57.1	.007	54.9	49	<.001	24.4	25.1	.23	15	12.9	<.001	11	12	.033
Some college	60.9	57	<.001	58.9	52.2	<.001	26.4	26.3	.88	15.9	13.6	<.001	12.1	12.6	.37
College	66.3	62.2	<.001	61.6	55.7	<.001	26.5	25.3	.02	16.8	14.1	<.001	10.9	10.6	.38
Insurance (age <65 y)															
Uninsured	34.1	29.4	.04	43.6	37.2	<.001	10.4	12.8	.02	5.7	5.1	.41	5.1	6.7	.03
Insured	62.2	58.2	<.001	60.5	54.2	<.001	24.2	24.7	.3	15.5	13	<.001	10	11	.008
Usual source of care															
No	31.32	28	.06	43.9	38.6	<.001	11	13.7	.002	5.9	6.3	.42	5.4	6.2	.28
Yes	64.45	60.8	<.001	61.9	55.5	<.001	27.5	27.5	1	16.9	14.2	<.001	12.3	13.1	.003

^a^ Other refers to race only, non-Hispanic, not otherwise specified.

**Table 4.** aPRs of Recent (Past-Year) Cancer Screening According to Sociodemographic, Socioeconomic, and Access to Care Variables, 2020 vs 2018

Variable	aPR (95% CI), 2020 vs 2018^a^				
	Breast	Cervical	Any CRC test	Colonoscopy	Stool test
Total	0.94 (0.92-0.95)	0.89 (0.87-0.91)	1.00 (0.98-1.03)	0.84 (0.82-0.87)	1.07 (1.02-1.12)
Sex					
Male	NA	NA	1.00 (0.96-1.04)	0.83 (0.79-0.88)	1.06 (0.99-1.13)
Female	0.94 (0.92-0.95)	0.89 (0.87-0.91)	1.01 (0.98-1.05)	0.86 (0.82-0.90)	1.08 (1.01-1.14)
Age category, y					
25-39	NA	0.89 (0.86-0.92)	NA	NA	NA
40-49	NA	0.88 (0.85-0.92)	NA	NA	NA
50-54	0.92 (0.88-0.96)	0.89 (0.84-0.95)	1.06 (0.99-1.13)	0.81 (0.74-0.87)	1.24 (1.09-1.40)
55-59	0.96 (0.92-1.00)	0.89 (0.83-0.95)	1.05 (0.99-1.13)	0.90 (0.83-0.98)	1.10 (0.98-1.24)
60-64	0.92 (0.88-0.95)	0.91 (0.86-0.96)	0.97 (0.92-1.03)	0.82 (0.77-0.88)	1.02 (0.92-1.12)
65-69	0.94 (0.91-0.97)	NA	0.99 (0.94-1.04)	0.84 (0.78-0.91)	1.05 (0.96-1.14)
70-75	0.96 (0.92-0.99)	NA	0.97 (0.92-1.02)	0.87 (0.81-0.94)	1.01 (0.93-1.09)
Race and ethnicity					
American Indian or Alaska Native	0.83 (0.73-0.95)	0.97 (0.83-1.14)	0.91 (0.76-1.08)	0.75 (0.59-0.95)	0.90 (0.68-1.19)
Asian or Pacific Islander	0.73 (0.60-0.88)	0.92 (0.81-1.05)	0.87 (0.70-1.09)	0.64 (0.47-0.88)	0.92 (0.66-1.29)
Hispanic	0.90 (0.83-0.97)	0.83 (0.77-0.88)	1.23 (1.11-1.36)	0.86 (0.74-1.01)	1.55 (1.34-1.79)
Non-Hispanic Black	0.97 (0.92-1.01)	0.93 (0.89-0.97)	1.01 (0.94-1.08)	0.89 (0.81-0.99)	1.00 (0.88-1.13)
Non-Hispanic White	0.95 (0.93-0.97)	0.89 (0.87-0.91)	0.98 (0.95-1.01)	0.85 (0.82-0.88)	1.01 (0.96-1.06)
Other^b^	0.85 (0.76-0.96)	0.94 (0.84-1.06)	0.83 (0.71-0.98)	0.67 (0.56-0.80)	0.88 (0.67-1.17)
Household income, $					
<25 000	0.93 (0.89-0.97)	0.87 (0.83-0.91)	1.12 (1.06-1.18)	0.89 (0.82-0.96)	1.24 (1.15-1.35)
25 to <75 000	0.91 (0.89-0.94)	0.89 (0.85-0.92)	1.00 (0.95-1.05)	0.86 (0.81-0.92)	1.07 (0.99-1.17)
≥75 000	0.95 (0.92-0.98)	0.90 (0.87-0.92)	0.96 (0.91-1.00)	0.85 (0.81-0.90)	1.00 (0.91-1.09)
Education					
Less than high school	0.90 (0.83-0.97)	0.83 (0.75-0.91)	1.16 (1.06-1.28)	0.83 (0.72-0.96)	1.39 (1.20-1.62)
High school	0.95 (0.92-0.99)	0.89 (0.85-0.93)	1.01 (0.97-1.06)	0.85 (0.80-0.91)	1.06 (0.98-1.15)
Some college	0.93 (0.90-0.96)	0.89 (0.86-0.92)	0.99 (0.94-1.04)	0.85 (0.80-0.91)	1.03 (0.95-1.12)
College	0.94 (0.91-0.96)	0.91 (0.88-0.93)	0.95 (0.92-0.99)	0.84 (0.80-0.88)	0.97 (0.90-1.05)
Insurance (age <65 y)					
Uninsured	0.86 (0.75-0.99)	0.85 (0.79-0.93)	1.25 (1.06-1.48)	0.94 (0.74-1.18)	1.32 (1.03-1.70)
Insured	0.93 (0.91-0.96)	0.90 (0.88-0.91)	1.02 (0.98-1.05)	0.84 (0.80-0.88)	1.10 (1.03-1.17)
Usual source of care					
No	0.89 (0.80-1.00)	0.88 (0.83-0.94)	1.23 (1.07-1.40)	1.06 (0.90-1.24)	1.12 (0.88-1.42)
Yes	0.94 (0.92-0.96)	0.90 (0.88-0.91)	1.00 (0.97-1.02)	0.84 (0.81-0.87)	1.07 (1.02-1.12)

Abbreviations: aPR, adjusted prevalence ratio; CRC, colorectal cancer; NA, not applicable.

^a^ Models were adjusted for age, sex (CRC screening), education, and state.

^b^ Other refers to race only, non-Hispanic, not otherwise specified.

Past-year BC screening prevalence decreased more among Hispanic (aPR, 0.90; 95% CI, 0.83-0.97), Asian (aPR, 0.73; 95% CI, 0.60-0.88), and American Indian or Alaska Native (aPR, 0.83; 95% CI, 0.73-0.95) women compared with White women (aPR, 0.95; 95% CI, 0.93-0.97) (Table 4). Decreases in past-year CC screening prevalence were also greater among Hispanic women (aPR, 0.83; 95% CI, 0.77-0.88) than White women (aPR, 0.89; 95% CI, 0.87-0.91) (Table 4). Among Asian persons, the decrease in colonoscopy prevalence (aPR, 0.64; 95% CI, 0.47-0.88) was greater than those for other racial and ethnic groups, and there was not a concomitant increase in stool testing; past-year CRC prevalence decreased in 2020 among Asian persons (aPR, 0.87; 95% CI, 0.70-1.09) but not significantly from 2018. Changes in self-reported past-year CRC screening prevalence for Black persons were not significantly different between 2018 and 2020 (aPR, 1.01; 95% CI, 0.94-1.08).

### Changes and Factors Associated With UTD Screening

Overall, the proportions of women reporting being UTD with BC and CC screening were similar between 2018 and 2020 (eTable 5 in the Supplement), a pattern that was consistent across race and ethnicity, education, and insurance status (eTable 6 in the Supplement). In a post hoc analysis, among women who were UTD with BC and CC screening, the proportion of women who received their screening tests within the past year was lower in 2020 compared with earlier years (eFigure 2 in the Supplement). For example, among women who were UTD with BC screening, 78.4% of 2020 respondents being screened in the past year compared with 78.9% in 2018.

The proportion of adults who were UTD with CRC screening increased by 4% between 2018 and 2020 (aPR, 1.04; 95% CI, 1.03-1.05), with greater increases among adults with lower educational attainment (aPR, 1.21; 95% CI, 1.15-1.26), Hispanic individuals (aPR, 1.16; 95% CI, 1.10-1.22), and uninsured adults (aPR, 1.15; 95% CI, 1.05-1.25) (eTable 5 in the Supplement). Yet, in 2020, people with lower educational attainment, Hispanic adults, and those who were uninsured were still less likely to be UTD with CRC cancer screening vs those with higher education, White adults, and insured persons (eTable 7 in the Supplement). Similar disparities in receipt of BC and CC screening were observed.

## Discussion

In this national survey study, past-year BC and CC screening prevalence decreased by 6% and 11%, respectively, between 2018 and 2020, and these decreases were consistently greater among people with lower educational attainment and Hispanic persons. Overall, there were an estimated 2.13 fewer million women who reported being recently screened for BC and 4.47 fewer million women screened for CC screening in 2020 than 2018. However, past-year CRC screening prevalence remained steady because decreases in colonoscopy use were offset by increases in stool testing.

Our finding that increased stool testing counterbalanced decreases in colonoscopy is important because it shows the realized potential of at-home testing to maintain population-wide screening rates during a major health care disruption. Commentaries^19,20^ hypothesized that increased stool tests could buffer against reported decreases in colonoscopy use during 2020, and our findings suggest that this appears to be the case. This finding also has implications for CC screening, where self-collected HPV sampling has been shown to improve screening uptake, although self-HPV sampling is not yet recommended because questions about its efficacy remain.^21,22^ The striking increases in recent stool testing among people with lower SES from 2018 to 2020 is an encouraging finding that may stem from growing use of stool testing outreach in health systems, including federally qualified health clinics (FQHCs) that serve lower-income populations.^23,24^ This is a strategy shown to boost CRC screening utilization.^23,24^ In our study, UTD CRC screening improved the most among people who had the least education, but despite these improvements, inequities in screening remain.

The recent increase in stool-based CRC screening heightens the importance of implementing evidence-based strategies (eg, patient navigation and tracking) to ensure that positive stool tests are promptly followed by colonoscopy, as stool testing is effective only when this recommended screening process is complete.^25,26,27,28^ This may be especially important in the populations (ie, people with the lower SES) for whom stool testing is commonly used. Before the pandemic, receipt of follow-up colonoscopy among people with positive stool tests was suboptimal, with reported rates of less than 50% among FQHCs.^29^ This finding also underscores the timeliness of a federal rule issued in early 2022 addressing patient out-of-pocket costs for a follow-up colonoscopy after a positive stool test.^30^ Previously, health insurers and Medicare were not required to fully pay for follow-up colonoscopy after a positive stool test because it was considered diagnostic, even though a follow-up colonoscopy is a necessary component of the screening process and all preventive services with A or B USPSTF recommendation are supposed to be completely cost-free under the Patient Protection and Affordable Care Act.

Our observation of population-wide decreases in recent BC and CC screening prevalence in 2020 compared with 2018, alongside studies of medical records and claims,^6,8,9^ may signal that the rebounds in screening volume beginning in the summer of 2020 may not have fully compensated for the near halt in screening during March and April. This may be especially true for Hispanic persons and people with lower SES as these groups experienced greater decreases in BC and CC screening,^5,9^ reflecting newly emerging and exacerbation of long-standing barriers to screening. This is concerning because these populations are less likely to be UTD with cancer screening and have higher death rates for some screen-detectable cancers.^31^ Early in the pandemic when routine procedures were paused, BC screening volumes decreased more sharply among Hispanic, Asian, and American Indian and Alaska Native persons, and recovery was also weaker in these groups.^32,33^ At least 1 FQHC experienced greater delays in fully resuming BC screening services compared with nearby academic centers,^34^ and other studies of FQHCs note that staff were diverted away from screening navigation and outreach during the pandemic.^35^ Less obvious structural factors may have created barriers; for example, health care systems may have prioritized those at higher risk of developing cancer, and risk-based models are often based on White populations.^34^ In addition, people cited fear of COVID-19 as a reason to avoid medical care, and people’s assessment of the benefits of preventive medical care vs COVID-19 risk may be influenced by COVID-19 burden in their community.^1^

Broader social and economic issues may have disproportionately affected cancer screening utilization during the pandemic. People with lower SES were more likely to lose employment, have reduced hours or wages, and have a worsening financial situation during the pandemic.^36,37^ Health insurance rates were unexpectedly steady during 2020 amid increasing unemployment rates. This may be partly because job losses were concentrated among low-wage workers who did not have employer-sponsored insurance to begin with, and, in 2020, there was enhanced Medicaid funding, and Marketplace and Medicaid Expansion enrollment periods were opened.^38,39,40^

In our study, decreases in recent CC and BC screening have not yet influenced the proportion of women UTD with these cancer screenings. Women screened more frequently than recommended may have maintained their UTD BC and CC screening status in 2020 despite not having a recent test. Previous studies^41,42^ have shown that physicians frequently recommend, and women often receive, BC and CC screening on an annual basis, even though the USPSTF, American Cancer Society, and professional organizations lengthened BC and CC screening intervals many years ago. It is possible that the pandemic may have reduced overuse of screening. Among women in our study who were UTD for BC and CC screening, the proportion who received their cancer screening tests within the past year was lower in 2020 compared with earlier years. What the potential lengthening of screening intervals and declines in recent BC and CC screening means for outcomes is not yet known. Modeling studies^43^ assuming a 6-month pause in mammography projected a relatively small number of excess BC deaths through 2030, and a 6-month pause in CC screening may lead to a slight stage shift, with minimal impacts on CC outcomes.^44^ A recent study^45^ of electronic pathology reports showed a 9% decrease in the number of BCs in 2020 compared with 2019, although the stage and outcomes of cancers diagnosed in 2020 and beyond is not yet known. Previous observational studies examining outcomes after the 2009 USPSTF recommendation to lengthen BC screening intervals for women aged 50 to 74 years to every 2 years showed no immediate changes in stage distribution,^46^ although screening intervals and practices also did not change.^42^ Ongoing study of dynamics of screening utilization throughout the pandemic and outcomes are needed.

The magnitude and timing of population-wide decreases in past-year BC and CC screening as well as colonoscopy use in our study were different than medical claims or regional health system data. In those studies,^3,4,5,6,7,8,9,47^ mammography, Papanicolaou and HPV testing, and colonoscopy volumes decreased by 70% to 90% in March and April of 2020 compared with expected volumes, and then approached expected volumes by summer or fall of 2020. In our study, decreases in self-reported screening behavior began in the third quarter (July-September) of 2020. This is likely due to how screening was ascertained because we examined self-reported past-year screening rates. For example, people asked about their most recent screening experience in April of 2020 could have been screened in 2019, whereas people interviewed in the latter part of 2020 would have a greater chance of reporting their 2020 experience. In addition, the BRFSS sample was national and not limited geographical area, health care network, or specific type of health insurance coverage.

### Limitations

Our study has several limitations. Overall BRFSS response rates were similar in 2018 and 2020, yet some states paused interviews during shutdowns, and the raw proportion of respondents with lower educational attainment decreased in 2020. Although we used population-based weights that accounted for nonresponse, biases may remain and pandemic-related changes in cancer screening prevalence may be underestimated, especially among people with lower educational attainment. Bias analyses of US Census surveys have noted greater nonresponse among people with lower incomes during 2020 than previous years, and ongoing efforts to improve data collection in vulnerable populations are needed.^48^ Furthermore, survey respondents tend be more healthy than the general population and it is unclear whether this worsened during the pandemic.^49^ Our data were self-reported and prone to recall biases, although a previous meta-analysis^50^ suggests that the overall accuracy of self-reported screening is good. An additional limitation is that we did not examine the reason for a test (eg, for routine screening vs a problem or diagnostic reasons).

## Conclusions

In a national survey study, past-year CRC screening prevalence remained level during 2020 because decreases in colonoscopy use were offset by an increase in stool testing, showing the promise of at-home testing to maintain population-wide screening rates during a major health care disruption. Meanwhile, past-year BC and CC screening prevalence decreased, especially among people with lower educational attainment and Hispanic persons, perhaps because of both newly emerging and existing barriers to health care. What these decreases in recent BC and CC screening mean for immediate and long-term outcomes is not yet known but will be important to monitor, especially among people with lower SES.

## Notes

Supplement. **eFigure 1.** Estimated Differences in Past-Year Cancer Screening in 2020 v 2018, BRFSS **eFigure 2.** Time Since Most Recent Screening Among People Up to Date With Breast, Cervical, and Colorectal Cancer Screening, BRFSS 2014-2020 **eTable 1.** Definitions of Cancer Screening and Other Variables **eTable 2.** Proportion of Interviews Conducted According to Month, BRFSS 2018 and 2020 **eTable 3.** Number of Respondents Excluded, BRFSS 2014, 2016, 2018, and 2020 **eTable 4.** Adjusted Prevalence Ratios of Recent Cancer Screening Among Interviews Conducted in April-December 2020 v April-December 2018 **eTable 5.** Adjusted Prevalence Ratios 2020 v 2018 of Up to Date Cancer Screening According to Sociodemographic, Health Insurance, and Health Care Factors **eTable 6.** Prevalence of Up-to-Date Breast, Cervical, Colorectal Cancer Screening in 2018 and 2020 **eTable 7.** Predictors of Up-to-Date Breast, Cervical, Colorectal Cancer Screening in 2020

## References

[1] Czeisler ME, Marynak K, Clarke KEN, . Delay or avoidance of medical care because of COVID-19-related concerns: United States, June 2020. MMWR Morb Mortal Wkly Rep. 2020;69(36):1250-1257. doi:10.15585/mmwr.mm6936a4 32915166 PMC7499838

[2] Mast C. Delayed cancer screenings—a second look. EPIC Health Research Network. July 17, 2020. Accessed April 27, 2022. https://epicresearch.org/articles/delayed-cancer-screenings-a-second-look

[3] Bakouny Z, Paciotti M, Schmidt AL, Lipsitz SR, Choueiri TK, Trinh QD. Cancer screening tests and cancer diagnoses during the COVID-19 pandemic. JAMA Oncol. 2021;7(3):458-460. doi:10.1001/jamaoncol.2020.7600 33443549 PMC7809614

[4] Nyante SJ, Benefield TS, Kuzmiak CM, Earnhardt K, Pritchard M, Henderson LM. Population-level impact of coronavirus disease 2019 on breast cancer screening and diagnostic procedures. Cancer. 2021;127(12):2111-2121. doi:10.1002/cncr.33460 33635541 PMC8013451

[5] DeGroff A, Miller J, Sharma K, . COVID-19 impact on screening test volume through the National Breast and Cervical Cancer early detection program, January-June 2020, in the United States. Prev Med. 2021;151:106559. doi:10.1016/j.ypmed.2021.106559 34217410 PMC9026719

[6] Chen RC, Haynes K, Du S, Barron J, Katz AJ. Association of cancer screening deficit in the United States with the COVID-19 pandemic. JAMA Oncol. 2021;7(6):878-884. doi:10.1001/jamaoncol.2021.0884 33914015 PMC8085759

[7] McBain RK, Cantor JH, Jena AB, Pera MF, Bravata DM, Whaley CM. Decline and rebound in routine cancer screening rates during the COVID-19 pandemic. J Gen Intern Med. 2021;36(6):1829-1831. doi:10.1007/s11606-021-06660-5 33742300 PMC7978463

[8] Song H, Bergman A, Chen AT, . Disruptions in preventive care: mammograms during the COVID-19 pandemic. Health Serv Res. 2021;56(1):95-101. doi:10.1111/1475-6773.13596 33146429 PMC7839639

[9] Sprague BL, Lowry KP, Miglioretti DL, . Changes in mammography use by women’s characteristics during the first 5 months of the COVID-19 pandemic. J Natl Cancer Inst. 2021;113(9):1161-1167. doi:10.1093/jnci/djab045 33778894 PMC8083761

[10] Goding Sauer A, Siegel RL, Jemal A, Fedewa SA. Current prevalence of major cancer risk factors and screening test use in the United States: disparities by education and race/ethnicity. Cancer Epidemiol Biomarkers Prev. 2019;28(4):629-642. doi:10.1158/1055-9965.EPI-18-1169 30944145

[11] Centers for Disease Control and Prevention. Behavioral Risk Factor Surveillance System 2020 summary data quality report. August 2, 2021. Accessed April 27, 2022. https://www.cdc.gov/brfss/annual_data/2020/pdf/2020-sdqr-508.pdf

[12] US Department of Health and Human Services Office for Human Research Protections. Human subject regulations. 2022. Accessed April 27, 2022. https://www.hhs.gov/ohrp/regulations-and-policy/index.html

[13] Klein RJ, Proctor SE, Boudreault MA, Turczyn KM. Healthy People 2010 criteria for data suppression. *Statistical Notes*. July 2002. Accessed May 3, 2022. https://citeseerx.ist.psu.edu/viewdoc/download?doi=10.1.1.200.3181&rep=rep1&type=pdf 12117004

[14] Grossman DC, Curry SJ, Owens DK, ; US Preventive Services Task Force. Screening for prostate cancer: US Preventive Services Task Force recommendation statement. JAMA. 2018;319(18):1901-1913. doi:10.1001/jama.2018.3710 29801017

[15] Hall IJ, Tangka FKL, Sabatino SA, Thompson TD, Graubard BI, Breen N. Patterns and trends in cancer screening in the United States. Prev Chronic Dis. 2018;15:E97. doi:10.5888/pcd15.170465 30048233 PMC6093265

[16] Hiatt RA, Klabunde C, Breen N, Swan J, Ballard-Barbash R. Cancer screening practices from National Health Interview Surveys: past, present, and future. J Natl Cancer Inst. 2002;94(24):1837-1846. doi:10.1093/jnci/94.24.1837 12488477

[17] Shapiro JA, Klabunde CN, Thompson TD, Nadel MR, Seeff LC, White A. Patterns of colorectal cancer test use, including CT colonography, in the 2010 National Health Interview Survey. Cancer Epidemiol Biomarkers Prev. 2012;21(6):895-904. doi:10.1158/1055-9965.EPI-12-0192 22490320 PMC4489134

[18] Bieler GS, Brown GG, Williams RL, Brogan DJ. Estimating model-adjusted risks, risk differences, and risk ratios from complex survey data. Am J Epidemiol. 2010;171(5):618-623. doi:10.1093/aje/kwp440 20133516

[19] Jaklevic MC. Pandemic spotlights in-home colon cancer screening tests. JAMA. 2021;352(2):116-118. doi:10.1001/jama.2020.22466 33355612

[20] Issaka RB, Somsouk M. Colorectal cancer screening and prevention in the COVID-19 era. JAMA Health Forum. 2020;1(5):e200588. doi:10.1001/jamahealthforum.2020.0588 34532717 PMC8443218

[21] Bishop E, Katz ML, Reiter PL. Acceptability of human papillomavirus self-sampling among a national sample of women in the United States. Biores Open Access. 2019;8(1):65-73. doi:10.1089/biores.2018.0040 31057989 PMC6497327

[22] Arbyn M, Smith SB, Temin S, Sultana F, Castle P; Collaboration on Self-Sampling and HPV Testing. Detecting cervical precancer and reaching underscreened women by using HPV testing on self samples: updated meta-analyses. BMJ. 2018;363:k4823. doi:10.1136/bmj.k4823 30518635 PMC6278587

[23] Gupta S, Coronado GD, Argenbright K, . Mailed fecal immunochemical test outreach for CRC screening: summary of a Centers for Disease Control and Prevention–sponsored summit. CA Cancer J Clin. 2020;70(4):283-298. doi:10.3322/caac.21615 32583884 PMC7523556

[24] Kemper KE, Glaze BL, Eastman CL, . Effectiveness and cost of multilayered colorectal cancer screening promotion interventions at federally qualified health centers in Washington State. Cancer. 2018;124(21):4121-4129. doi:10.1002/cncr.31693 30359468 PMC6263795

[25] Selby K, Baumgartner C, Levin TR, . Interventions to improve follow-up of positive results on fecal blood tests: a systematic review. Ann Intern Med. 2017;167(8):565-575. doi:10.7326/M17-1361 29049756 PMC6178946

[26] Krist AH, Davidson KW, Mangione CM, ; US Preventive Services Task Force. Screening for lung cancer: US Preventive Services Task Force recommendation statement. JAMA. 2021;325(10):962-970. doi:10.1001/jama.2021.1117 33687470

[27] Corley DA, Jensen CD, Quinn VP, . Association between time to colonoscopy after a positive fecal test result and risk of colorectal cancer and cancer stage at diagnosis. JAMA. 2017;317(16):1631-1641. doi:10.1001/jama.2017.3634 28444278 PMC6343838

[28] San Miguel Y, Demb J, Martinez ME, Gupta S, May FP. Time to colonoscopy after abnormal stool-based screening and risk for colorectal cancer incidence and mortality. Gastroenterology. 2021;160(6):1997-2005.e3. doi:10.1053/j.gastro.2021.01.219 33545140 PMC8096663

[29] Bharti B, May FFP, Nodora J, . Diagnostic colonoscopy completion after abnormal fecal immunochemical testing and quality of tests used at 8 Federally Qualified Health Centers in Southern California: Opportunities for improving screening outcomes. Cancer. 2019;125(23):4203-4209. doi:10.1002/cncr.32440 31479529 PMC7008958

[30] US Department of Labor. FAQs about Affordable Care Act implementation part 51, families first coronavirus response act and coronavirus aid, relief, and economic security act implementation. January 10, 2022. Accessed March 9, 2022. https://www.dol.gov/sites/dolgov/files/EBSA/about-ebsa/our-activities/resource-center/faqs/aca-part-51.pdf

[31] Singh GK, Jemal A. Socioeconomic and racial/ethnic disparities in cancer mortality, incidence, and survival in the United States, 1950-2014: over six decades of changing patterns and widening inequalities. J Environ Public Health. 2017;2017:2819372. doi:10.1155/2017/2819372 28408935 PMC5376950

[32] Anderson KE, McGinty EE, Presskreischer R, Barry CL. Reports of forgone medical care among US adults during the initial phase of the COVID-19 pandemic. JAMA Netw Open. 2021;4(1):e2034882. doi:10.1001/jamanetworkopen.2020.34882 33475757 PMC7821029

[33] Amram O, Robison J, Amiri S, Pflugeisen B, Roll J, Monsivais P. Socioeconomic and racial inequities in breast cancer screening during the COVID-19 pandemic in Washington State. JAMA Netw Open. 2021;4(5):e2110946. doi:10.1001/jamanetworkopen.2021.10946 34028552 PMC8144923

[34] Lehman CD, Mercaldo SF, Wang GX, Dontchos BN, Specht MC, Lamb LR. Screening mammography recovery after COVID-19 pandemic facility closures: associations of facility access and racial and ethnic screening disparities. AJR Am J Roentgenol. Published online March 30, 2022. doi:10.2214/AJR.21.26890 34817192

[35] Fisher-Borne M, Isher-Witt J, Comstock S, Perkins RB. Understanding COVID-19 impact on cervical, breast, and colorectal cancer screening among federally qualified healthcare centers participating in “Back on track with screening” quality improvement projects. Prev Med. 2021;151:106681. doi:10.1016/j.ypmed.2021.106681 34217422 PMC8241686

[36] Harvard TH Chan School of Public Health; Robert Wood Johnson Foundation. Household experiences in America during the Delta variant outbreak, by race/ethnicity. October 2021. Accessed April 27, 2022. https://cdn1.sph.harvard.edu/wp-content/uploads/sites/94/2021/10/EthnicityRWJFNPRHORP.pdf

[37] Brown S. How COVID-19 is affecting Black and Latino families’ employment and financial well-being. Urban Institute. May 6, 2020. Accessed April 27, 2022. https://www.urban.org/urban-wire/how-covid-19-affecting-black-and-latino-families-employment-and-financial-well-being

[38] Tolbert J, Orgera K, Damico A. What does the CPS tell us about health insurance coverage in 2020? Kaiser Family Foundation. September 23, 2021. Accessed December 2, 2021. https://www.kff.org/uninsured/issue-brief/what-does-the-cps-tell-us-about-health-insurance-coverage-in-2020/

[39] Corallo B, Garfield R, Tolbert J, Rudowitz R. Medicaid enrollment churn and implications for continuous coverage policies. Kaiser Family Foundation. December 14, 2021. Accessed January 5, 2022. https://www.kff.org/medicaid/issue-brief/medicaid-enrollment-churn-and-implications-for-continuous-coverage-policies

[40] Ruhter J, Conmy AB, Chu RC, Peters C, De Lew N, Sommers BD. Tracking health insurance coverage in 2020-2021. Office of the Assistant Secretary of Planning and Evaluation, US Department of Health and Human Services. October 29, 2021. Accessed December 2, 2021. https://aspe.hhs.gov/sites/default/files/documents/2fb03bb1527d26e3f270c65e2bfffc3a/tracking-insurance-coverage-2020-2021.pdf

[41] Haas JS, Sprague BL, Klabunde CN, ; PROSPR Consortium. Provider attitudes and screening practices following changes in breast and cervical cancer screening guidelines. J Gen Intern Med. 2016;31(1):52-59. doi:10.1007/s11606-015-3449-5 26129780 PMC4700005

[42] Wernli KJ, Arao RF, Hubbard RA, . Change in breast cancer screening intervals since the 2009 USPSTF guideline. J Womens Health (Larchmt). 2017;26(8):820-827. doi:10.1089/jwh.2016.6076 28177856 PMC5576213

[43] Alagoz O, Lowry KP, Kurian AW, ; from the CISNET Breast Working Group. Impact of the COVID-19 pandemic on breast cancer mortality in the US: estimates from collaborative simulation modeling. J Natl Cancer Inst. 2021;113(11):1484-1494. doi:10.1093/jnci/djab097 34258611 PMC8344930

[44] Burger EA, Jansen EE, Killen J, . Impact of COVID-19-related care disruptions on cervical cancer screening in the United States. J Med Screen. 2021;28(2):213-216. doi:10.1177/09691413211001097 33730899 PMC8484743

[45] Yabroff KR, Wu XC, Negoita S, . Association of the COVID-19 pandemic with patterns of statewide cancer services. J Natl Cancer Inst. 2021;djab122. doi:10.1093/jnci/djab122 34181001 PMC9194624

[46] Guo F, Kuo YF, Berenson AB. Breast cancer incidence by stage before and after change in screening guidelines. Am J Prev Med. 2019;56(1):100-108. doi:10.1016/j.amepre.2018.08.018 30573138 PMC6312406

[47] Miller MJ, Xu L, Qin J, . Impact of COVID-19 on cervical cancer screening rates among women aged 21-65 years in a large integrated health care system: Southern California, January 1-September 30, 2019, and January 1-September 30, 2020. MMWR Morb Mortal Wkly Rep. 2021;70(4):109-113. doi:10.15585/mmwr.mm7004a1 33507893 PMC7842810

[48] Rothbaum J, Bee A. Coronavirus infects surveys, too: survey nonresponse bias and the coronavirus pandemic. US Census Bureau. March 30, 2021. Accessed April 27, 2022. https://www.census.gov/library/working-papers/2020/demo/SEHSD-WP2020-10.html

[49] Keyes KM, Rutherford C, Popham F, Martins SS, Gray L. How healthy are survey respondents compared with the general population? using survey-linked death records to compare mortality outcomes. Epidemiology. 2018;29(2):299-307. doi:10.1097/EDE.0000000000000775 29389712 PMC5794231

[50] Rauscher GH, Johnson TP, Cho YI, Walk JA. Accuracy of self-reported cancer-screening histories: a meta-analysis. Cancer Epidemiol Biomarkers Prev. 2008;17(4):748-757. doi:10.1158/1055-9965.EPI-07-2629 18381468

