# Supplementary material for: Changes in Cancer Screening in the US During the COVID-19 Pandemic
Source: JAMA Netw Open. 2022 Jun 3;5(6):e2215490. doi: 10.1001/jamanetworkopen.2022.15490 (PMC9166223; doi:10.1001/jamanetworkopen.2022.15490)
Supplement: Supplement. — eFigure 1. Estimated Differences in Past-Year Cancer Screening in 2020 v 2018, BRFSS eFigure 2. Time Since Most Recent Screening Among People Up to Date With Breast, Cervical, and Colorectal Cancer Screening, BRFSS 2014-2020 eTable 1. Definitions of Cancer Screening and Other Variables eTable 2. Proportion of Interviews Conducted According to Month, BRFSS 2018 and 2020 eTable 3. Number of Respondents Excluded, BRFSS 2014, 2016, 2018, and 2020 eTable 4. Adjusted Prevalence Ratios of Recent Cancer Screening Among Interviews Conducted in April-December 2020 v April-December 2018 eTable 5. Adjusted Prevalence Ratios 2020 v 2018 of Up to Date Cancer Screening According to Sociodemographic, Health Insurance, and Health Care Factors eTable 6. Prevalence of Up-to-Date Breast, Cervical, Colorectal Cancer Screening in 2018 and 2020 eTable 7. Predictors of Up-to-Date Breast, Cervical, Colorectal Cancer Screening in 2020 [file jamanetwopen-e2215490-s001.pdf]

Supplemental Online Content

Fedewa SA, Star J, Bandi P, et al. Changes in cancer screening in the US during the COVID-19 pandemic. *JAMA Netw Open*. 2022;5(6):e2215490. doi:10.1001/jamanetworkopen.2022.15490

eFigure 1. Estimated Differences in Past-Year Cancer Screening in 2020 v 2018, BRFSS

eFigure 2. Time Since Most Recent Screening Among People Up to Date With Breast, Cervical, and Colorectal Cancer Screening, BRFSS 2014-2020

eTable 1. Definitions of Cancer Screening and Other Variables

eTable 2. Proportion of Interviews Conducted According to Month, BRFSS 2018 and 2020

eTable 3. Number of Respondents Excluded, BRFSS 2014, 2016, 2018, and 2020

eTable 4. Adjusted Prevalence Ratios of Recent Cancer Screening Among Interviews Conducted in April-December 2020 v April-December 2018

eTable 5. Adjusted Prevalence Ratios 2020 v 2018 of Up to Date Cancer Screening According to Sociodemographic, Health Insurance, and Health Care Factors

eTable 6. Prevalence of Up-to-Date Breast, Cervical, Colorectal Cancer Screening in 2018 and 2020

eTable 7. Predictors of Up-to-Date Breast, Cervical, Colorectal Cancer Screening in 2020

This supplemental material has been provided by the authors to give readers additional information about their work.

© 2022 Fedewa SA et al. *JAMA Network Open.*

eFigure 1. Estimated Differences in Past-Year Cancer Screening in 2020 v 2018, BRFSS[a]

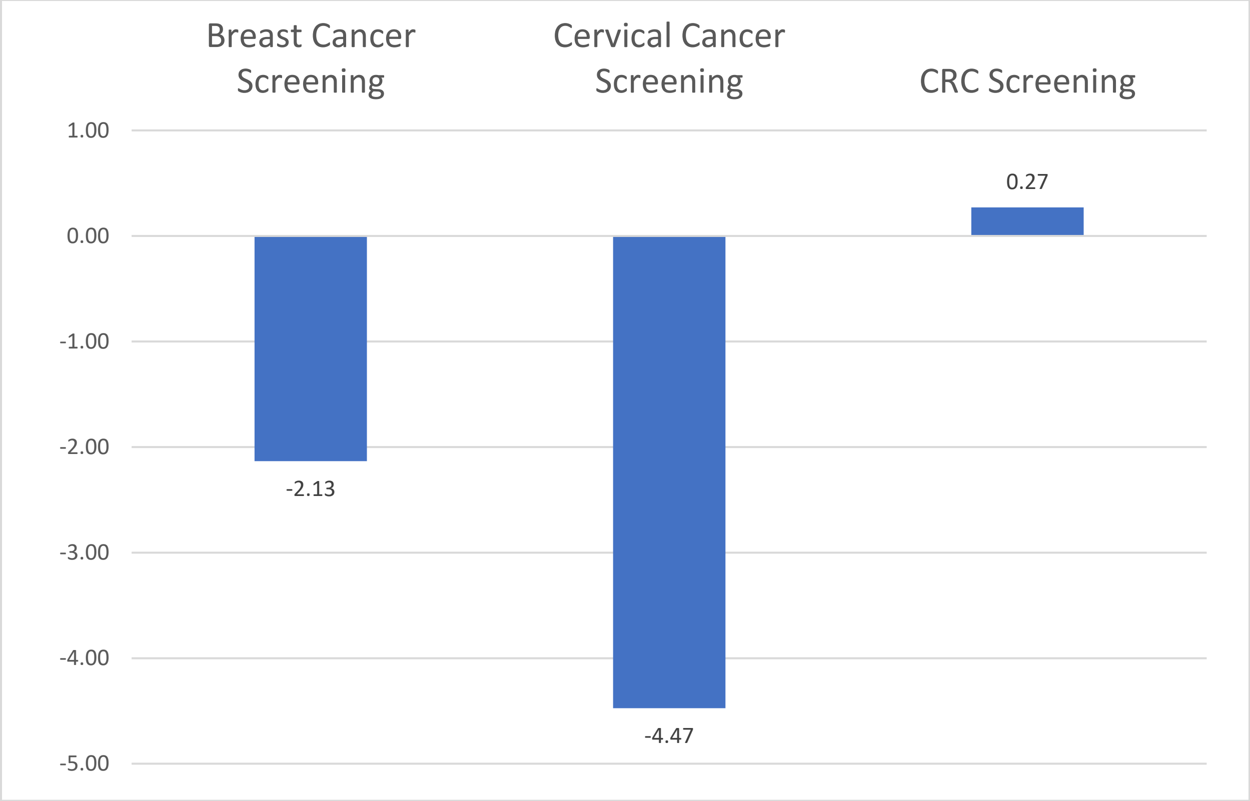

a. Deficits were computed using BRFSS weighted sums in 2018 v 2020

© 2022 Fedewa SA et al. *JAMA Network Open.*

eFigure 2. Time Since Most Recent Screening Among People Up to Date With Breast, Cervical, and Colorectal Cancer Screening, BRFSS 2014-2020[a]

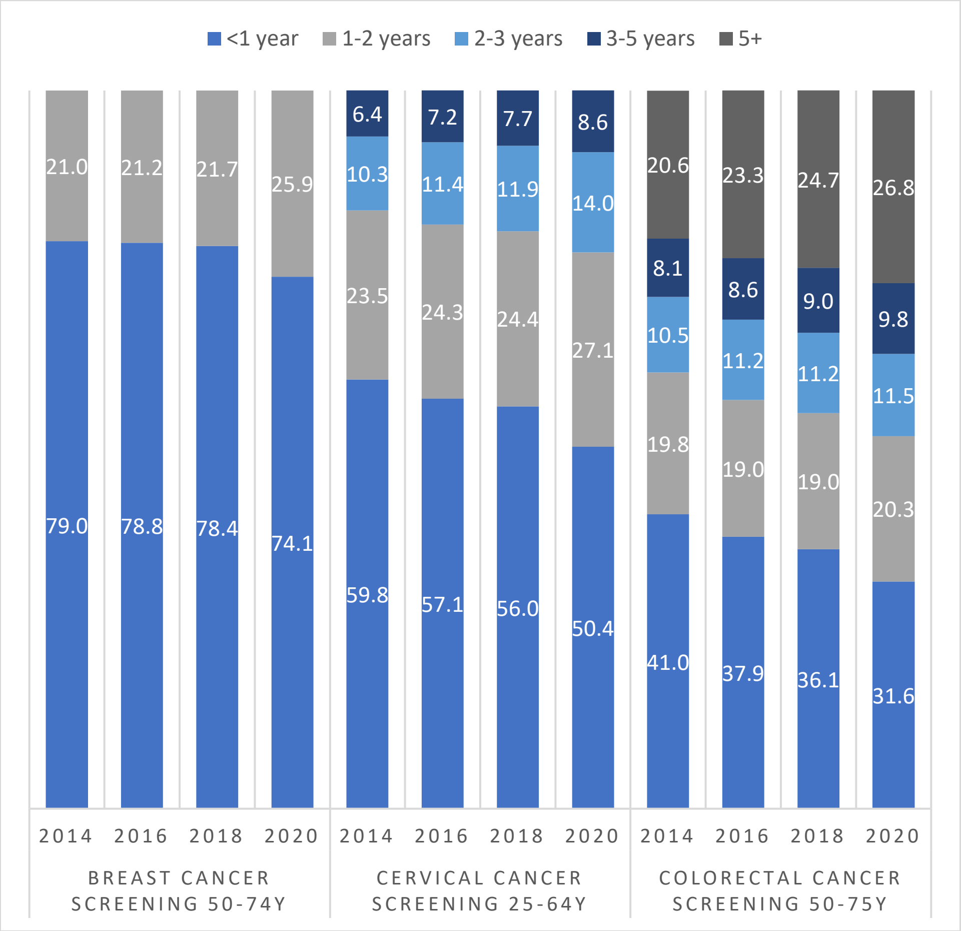

a. Up to date measures are defined in eTable 1

© 2022 Fedewa SA et al. *JAMA Network Open.*

eTable 1. Definitions of Cancer Screening and Other Variables

BRFSS Interview Questions	Response options
For each cancer screening test, respondents are asked:<br>Have you ever had a [fill in] test?<br>Tests included:<br>Breast: mammogram<br>Cervical testing, HPV testing (beginning in the 2016 survey)<br>Colorectal cancer[a]: colonoscopy, sigmoidoscopy, stool-testing, sDNA (beginning in the 2020 survey), CT colonography (beginning in the 2020 survey)	Yes<br>No
How long has it been since your most recent test?	Within the past year (anytime less than 12 months ago)<br>Within the past 2 years (1 year but less than 2 years ago)<br>Within the past 5 years (2 years but less than 5 years ago)<br>Within the past 10 years (5 years but less than 10 years ago)<br>10 or more years ago<br>Don't know
**Outcome/Variable**	**Definition**
Up to Date Cervical	Respondents with a Pap test in the past three years and beginning in 2016, respondents reporting a HPV plus Pap-testing (co-testing) in the past five years were considered to be up to date as this was the first time BRFSS included co-testing questions since it was recommended in 2012.[48]
Up to Date Breast	Respondents reporting a mammogram in the past two years
Up to Date Colorectal	Respondents reporting a fecal immunochemical test (FIT) or fecal occult blood test (FOBT) in the past year, sigmoidoscopy in the past five years, or colonoscopy in the past 10 years. Beginning in 2020, respondents reporting a sDNA in the past three years or CT Colonography were considered to be up to date as this was the first time BRFSS included these questions.

a. For colorectal cancer screening: respondents were asked if they had received a Sigmoidoscopy or a Colonoscopy and which they had received more recently. In 2020, respondents were asked if they had sigmoidoscopy and they were also asked if they had a colonoscopy

© 2022 Fedewa SA et al. *JAMA Network Open.*

eTable 2. Proportion of Interviews Conducted According to Month, BRFSS 2018 and 2020[a]

Month	% Interviewed in 2018	% Interviewed in 2020
January	6.7	4.5
February	9.2	8.0
March	9.3	11.2
April	8.1	9.0
May	7.7	8.8
June	8.2	8.0
July	8.5	7.9
August	8.2	8.2
September	8.2	7.4
October	9.2	8.3
November	8.6	9.8
December	8.1	9.0

a. The following states did not begin to collect data until February: Idaho, Maine, Michigan, New Mexico, North Dakota, Pennsylvania, South Carolina, South Dakota, Tennessee, Utah, and Virginia. California began data collection in March. Kentucky and New Jersey began data collection in May. Louisiana began data collection in June. 32 states did not complete their interviews by the end of 2020 and collected data in early 2021. We did not include respondents interviewed in 2021 in our quarterly estimates.

eTable 3. Number of Respondents Excluded, BRFSS 2014, 2016, 2018, and 2020

	**Breast Cancer Screening**	**Cervical Cancer Screening**	**Colorectal Cancer Screening**
**Age Ranges included**	**50-74 years**	**25-64 years**	**50-75 years**
No. of age-eligible and sex eligible respondents	511,467	581,826	932,042
No. and % missing screening data	32,219 (6.3%)	47,013 (8.1%)	77,832 (8.4%)
No. who had a hysterectomy or were missing data	--	175,256	--
**No. included in unadjusted analyses**	479,248	301,453	854,210
No. and % missing education	2,548 (0.5%)	2,717 (0.9%)	4,700 (0.6%)
No. included in adjusted analyses	476,700	298,736	849,510

Abbreviations Number (No)

© 2022 Fedewa SA et al. *JAMA Network Open.*

eTable 4. Adjusted Prevalence Ratios of Recent Cancer Screening Among Interviews Conducted in April-December 2020 v April-December 2018

	Breast (Women 50-74y)			Cervical (Women 25-64y)			Any CRC Test (Adults 50-75y)			Colonoscopy (Adults 50-75y)			Stool Testing (Adults 50-75y)		
Past-year Testing	aPR[a]	95%CI		aPR[a]	95%CI		aPR[a]	95%CI		aPR[a]	95%CI		aPR[a]	95%CI	
April- Dec 2020 v 2018	0.92	0.90	0.94	0.88	0.86	0.90	0.99	0.96	1.02	0.84	0.81	0.88	1.04	0.98	1.09
Up to Date	aPR[a]	95%CI		aPR[a]	95%CI		aPR[a]	95%CI		aPR[a]	95%CI		aPR[a]	95%CI	
April-Dec 2020 v 2018	0.99	0.98	1.01	0.98	0.97	0.99	1.04	1.03	1.05	0.99	0.98	1.00	1.12	1.08	1.16

Abbreviations: Year (y); Adjusted prevalence ratio (aPR); Confidence Interval (CI)

a. Models adjusted for state, education, age, and sex (CRC screening only)

© 2022 Fedewa SA et al. *JAMA Network Open.*

eTable 5. Adjusted Prevalence Ratios 2020 v 2018 of Up to Date Cancer Screening According to Sociodemographic, Health Insurance, and Health Care Factors

	Breast Cancer Screening(50-74 years)	Cervical Cancer Screening (25-65 years)	CRC Screening Prevalence (50-75 years)
	**aPR[a](95%CI)**	**aPR[a](95%CI)**	**aPR[a](95%CI)**
**TOTAL**	0.99(0.98, 1.00)	0.98(0.97, 0.99)	1.04(1.03, 1.05)
**Sex**			
Male	--	--	1.05(1.03, 1.06)
Female	--	--	1.03(1.01, 1.04)
**Age Category**			
25-39	--	0.97(0.95, 0.98)	--
40-49	--	0.98(0.96, 1.00)	--
50-54	0.99(0.96, 1.02)	0.98(0.95, 1.01)	1.08(1.05, 1.12)
55-59	1.02(0.99, 1.05)	0.99(0.97, 1.02)	1.03(1.01, 1.06)
60-64	0.98(0.96, 1.00)	0.98(0.95, 1.01)	1.02(1.00, 1.04)
65-69	0.98(0.96, 1.00)		1.03(1.01, 1.05)
70-75	0.98(0.96, 1.01)	--	1.03(1.01, 1.04)
**Race Category**			
AI/AN	0.91(0.84, 1.00)	1.02(0.94, 1.10)	0.99(0.91, 1.08)
Asian/PI	0.93(0.82, 1.05)	0.95(0.88, 1.02)	0.94(0.85, 1.05)
Hispanic	1.01(0.96, 1.06)	0.95(0.92, 0.98)	1.16(1.10, 1.22)
NH Black	1.01(0.98, 1.04)	0.99(0.97, 1.01)	1.06(1.02, 1.09)
NH White	0.99(0.98, 1.00)	0.98(0.97, 0.99)	1.02(1.01, 1.03)
Other[b]	0.92(0.85, 1.00)	0.98(0.92, 1.04)	1.00(0.93, 1.07)
**Income Category**			
<25,000	0.98(0.96, 1.01)	0.96(0.94, 0.99)	1.11(1.08, 1.14)
25-<75,000	0.99(0.97, 1.01)	0.97(0.95, 0.99)	1.02(1.00, 1.04)
>=75,000	0.99(0.97, 1.01)	0.99(0.98, 1.00)	1.00(0.98, 1.01)
**Education**			
<HS	1.02(0.97, 1.07)	0.93(0.89, 0.98)	1.21(1.15, 1.26)
HS	0.99(0.97, 1.01)	0.99(0.96, 1.01)	1.04(1.02, 1.06)
Some College	0.99(0.97, 1.01)	0.98(0.96, 0.99)	1.02(1.00, 1.04)
College	0.99(0.97, 1.00)	0.98(0.97, 0.99)	1.00(0.99, 1.01)
**Insurance (<65 years)**			
Uninsured	0.98(0.89, 1.07)	0.96(0.92, 1.00)	1.15(1.05, 1.25)
Insured	1.00(0.98, 1.01)	0.98(0.97, 0.99)	1.04(1.02, 1.05)
**PCP visits**			
PCP last year	0.99(0.98, 1.01)	0.98(0.97, 0.99)	1.05(1.04, 1.06)
No PCP last year	1.05(0.99, 1.10)	0.99(0.96, 1.02)	1.01(0.97, 1.05)
**Usual Source of Care**			

© 2022 Fedewa SA et al. *JAMA Network Open.*

Yes	0.99(0.98, 1.00)	0.98(0.97, 0.98)	1.03(1.02, 1.04)
No	1.00(0.93, 1.09)	0.99(0.96, 1.02)	1.10(1.04, 1.17)

© 2022 Fedewa SA et al. *JAMA Network Open.*

eTable 6. Prevalence of Up-to-Date Breast, Cervical, Colorectal Cancer Screening in 2018 and 2020[a]

	Breast (Women 50-74y)		Cervical (Women 25-64y)		UTD CRC Test (Adults 50-75y)		Colonoscopy – Ten Year (Adults 50-75y)		Stool Testing – Three Year (Adults 50-75y)	
	2018 (%)	2020 (%)	2018 (%)	2020 (%)	2018 (%)	2020 (%)	2018 (%)	2020 (%)	2018 (%)	2020 (%)
Total	78.9	78.4	82.1	80.2	69.3	72.4	63.9	63.3	18.6	21.3
Race/Ethnicity										
AI/AN	74.9	68.7	75.5	77.8	61.2	62.8	55.5	52.9	18.0	22.0
Asian/PI	78.8	72.7	74.6	70.0	65.5	61.1	56.5	46.6	24.5	26.7
Hispanic	79.4	79.9	83.2	78.9	54.8	63.9	47.9	48.0	19.3	31.2
NH Black	84.1	84.9	87.2	86.4	69.7	74.9	63.9	67.0	22.2	23.5
NH White	78.1	77.8	81.7	80.6	72.0	74.5	67.2	66.8	17.6	18.9
Other	76.5	69.7	79.0	75.9	65.3	65.1	59.0	55.2	23.0	20.8
Education										
<HS	73.3	74.5	75.4	70.2	53.2	64.1	46.3	48.6	18.5	27.4
HS	77.0	76.2	77.1	76.2	65.9	69.4	60.4	60.9	17.9	20.8
Some College	78.6	77.9	82.6	80.6	72.0	74.1	66.6	65.3	19.8	21.8
College	83.3	82.3	86.8	85.1	76.5	76.6	71.7	69.2	17.9	18.8
Insurance (<65y)										
Uninsured	52.7	51.4	66.9	64.1	32.1	36.5	28.3	28.8	8.9	11.9
Insured	79.7	79.7	84.4	82.8	67.0	70.0	61.7	60.9	16.3	19.1
Usual Source of Care										
No	48.3	49.1	67.6	66.8	34.4	38.4	30.4	31.5	9.5	11.6
Yes	81.8	81.3	85.8	83.8	73.7	76.7	68.2	67.3	19.7	22.6

Abbreviations: American Indian (AI); Alaskan Native (AN); High school (HS); Non-Hispanic (NH); up to date (UTD)

© 2022 Fedewa SA et al. *JAMA Network Open.*

eTable 7. Predictors of Up-to-Date Breast, Cervical, Colorectal Cancer Screening in 2020[a]

	CRC Screening (50-75y)	Breast Cancer Screening (50-74y)	Cervical Cancer Screening (25-64y)
	**aPR[a](95%CI)**	**aPR[a](95%CI)**	**aPR[a](95%CI)**
**Sex**			
Male	1.00		--
Female	1.02(1.01, 1.04)		--
**Age Category**			
25-39	--	--	1.08(1.05, 1.11)
40-49	--	--	1.07(1.04, 1.10)
50-54	0.68(0.66, 0.70)	0.93(0.91, 0.96)	1.07(1.03, 1.11)
55-59	0.88(0.86, 0.90)	0.96(0.94, 0.99)	1.04(1.01, 1.07)
60-64	0.93(0.91, 0.95)	0.97(0.95, 0.99)	1.00
65-69	1.00	1.00	--
70-75	1.05(1.03, 1.07)	1.00(0.98, 1.03)	--
**Race Category**			
AI/AN	0.91(0.85, 0.98)	0.93(0.84, 1.01)	1.00(0.96, 1.05)
Asian/PI	0.87(0.80, 0.95)	0.91(0.82, 1.02)	0.80(0.75, 0.86)
Hispanic	0.98(0.95, 1.01)	1.07(1.04, 1.11)	1.02(0.99, 1.04)
NH Black	1.04(1.01, 1.07)	1.11(1.08, 1.13)	1.08(1.06, 1.10)
NH White	1.00	1.00	1.00
Other[b]	0.91(0.86, 0.96)	0.91(0.84, 0.98)	0.93(0.88, 0.99)
**Income**			
<25,000	0.88(0.86, 0.91)	0.85(0.82, 0.87)	0.88(0.86, 0.90)
25-<75,000	0.93(0.91, 0.94)	0.93(0.91, 0.95)	0.93(0.91, 0.95)
>=75,000	1.00	1.00	1.00
**Education**			
<HS	0.86(0.83, 0.89)	0.91(0.87, 0.94)	0.82(0.79, 0.86)
HS	0.89(0.87, 0.91)	0.92(0.90, 0.94)	0.90(0.88, 0.92)
Some College	0.96(0.94, 0.97)	0.95(0.93, 0.97)	0.95(0.94, 0.97)
College	1.00	1.00	1.00
**Insurance Status[c]**			
Uninsured	0.58(0.55, 0.62)	0.66(0.61, 0.71)	0.80(0.78, 0.83)
Insured	1.00	1.00	1.00
**PCP visits (No vs Yes)**	0.63(0.61, 0.65)	0.65(0.63, 0.68)	0.75(0.73, 0.77)
**Usual Source of Care (No v Yes)**	0.58(0.55, 0.60)	0.62(0.58, 0.66)	0.80(0.78, 0.82)

a. Models adjusted for age, sex (CRC screening), race/ethnicity, education, usual provider, PCP visit.
b. Other race only, non-Hispanic
c. Models only include adults <65 years

© 2022 Fedewa SA et al. *JAMA Network Open.*
